# 

**Published:** 1882-09

**Authors:** 


					﻿N O T I C E.
All articles for publication, books for review, business communications, etc.,
must be sent to Editor, 2109 Chestnut Street, Philadelphia.
CONTRIBUTORS:
Drs. II. C> Allen, T. F. Alien, Edward Bayard, J, B. Bell; E. W. Perridge,
Geo. H. Clarke A.- C. Cowperthwaite, Wm. J. Guernsey, W. A. Hawley,
John Hall, W. M, James, W. H. Jenny, Ad. Lippe, C. Lippe,
Malcolm MacFarlan, C. F. Nichols, C. Pearson, T. F. Pom-
. erov, Leila A. RenDell, C. Carleton Smith, P. P. Wells,
Wm. P. Wesselhoeft, David Wilson (London).
T. P. Wilson, and many others.
Authors of accepted papers are. furnished with copies -or the
number in which their articles appear; application' for such copies
to be made when sending papers. Any irregularity ip the receipt
ofThis Journal should be promptly reported to Editor.
				

